# ADDIE-guided development and pilot feasibility evaluation of a prototype VR training simulation for suicide prevention among rural U.S. veterans

**DOI:** 10.1371/journal.pone.0345819

**Published:** 2026-08-24

**Authors:** Donna L. Schuman, Micki Washburn, Regina T. Praetorius, Jaci S. Mester, Minjaal Raval, Joshua Wilson, Suzanne Lohman, Renee Castillo

**Affiliations:** 1 School of Social Work, University of Texas at Arlington, Arlington, Texas, United States of America; 2 College of Nursing and Health Innovation, University of Texas at Arlington, Arlington, Texas, United States of America; 3 Department of Psychology, Texas Christian University, Fort Worth, Texas, United States of America; Taipei Veterans General Hospital, TAIWAN

## Abstract

**Background:**

Suicide risk is disproportionately higher for rural veterans who often lack access to specialty-trained health professionals equipped to deliver effective prevention interventions. Virtual reality offers a scalable training solution to address these gaps. This mixed-methods feasibility study systematically developed and evaluated a prototype virtual reality simulation for a training module in suicide risk identification and lethal means safety counseling during rural veteran home visits.

**Methods:**

Thirty participants (10 nursing/social work students, 10 health professionals, and 10 rural community members) completed the veteran suicide prevention simulation using Meta Quest 2 headsets. Sense of presence (including negative effects) was assessed with the ITC-Sense of Presence Inventory; additional outcomes included representativeness, acceptability, and cue recall.

**Results:**

Participants reported high engagement, with moderate spatial presence, and ecological validity, and low negative effects (e.g., nausea). Prominent lethal means cues were highly recalled (firearms: 93.3%; alcohol: 83.3%), while subtler cues (e.g., isolation) were less frequently noted. Most endorsed representational accuracy, with qualitative feedback praising authenticity and suggesting improvements in interactivity and navigation.

**Conclusions:**

Findings support the preliminary feasibility and acceptability of the VET-SAVR prototype for suicide risk identification and lethal means counseling training. Refinements to navigation, cue salience, and mitigation of negative VR effects are warranted before future efficacy testing.

## Introduction

Veterans have higher suicide rates than non-veterans. Based on 2022 data, age-adjusted rates are more than 44% higher for males and more than 92% higher for females [1]. Rates are even higher among the approximately 4.7 million veterans living in rural areas of the U.S. [2].

### Suicide risk and training gaps among rural veterans

Multiple intersecting factors contribute to rural veterans’ suicide risk. In addition to suicide risk factors impacting veterans regardless of geography (e.g., exposure to combat atrocities, posttraumatic stress disorder, depression, substance misuse, traumatic brain injury, chronic pain, military-civilian transition, help-seeking stigma, and firearm access [2–7]), rural veterans face greater social, economic, and health challenges that heighten their risk above that of urban veterans [2].

Suicide risk factors disproportionately impacting rural veterans include geographic isolation, higher rates of disability, lower socioeconomic status, self-reliance norms, mistrust of urban or national services (e.g., hotlines), and higher firearm ownership rates [8–11]. Firearms are also more often the method of suicide among rural than urban VA patients (76.8% vs 61.5%), and rural patients show roughly 22% greater suicide risk than urban patients even after accounting for mental health service accessibility [11]. This elevated case fatality highlights the specific need for lethal means safety in rural contexts. These challenges are compounded by veterans’ own barriers to seeking mental health care, including “the double-edged sword of intersecting stigmas,” referring to fears of being perceived as weak for seeking mental health support and as dangerous due to veteran status, amplified by rural values of rugged individualism ([9], p. 592).

Suicide attempts with firearms are far more likely to be fatal than other methods, with a case fatality rate of almost 90% [12]. This high lethality makes effective lethal means counseling especially urgent in rural areas, where many communities lack specialized behavioral health professionals, forcing rural veterans to depend on primary care providers and nurses for mental health services [13]. Research highlights severe shortages of suicide prevention resources tailored for rural veterans [14]. Critically, professionals report gaps in training and a desire for additional training in suicide risk assessment and suicide prevention/intervention [9,15].

### Evidence-based suicide prevention strategies

Culturally appropriate models that reflect rural veterans’ values stand the best chance of effectively preventing suicide in this population [9,16]. A proven example is lethal means safety planning, which is an evidence-based model that encourages culturally sensitive and respectful conversations about lethal means (i.e., objects, tools, or methods used in suicide; [17]). Lethal means safety planning, including secure storage of firearms and medications, can create lifesaving delays between impulses and access [18,19]. This approach resonates with rural veterans [20], particularly those who value firearms for protection, as it prioritizes safety over permanent loss of access, making it especially vital given that firearms are the most lethal method of self-harm, with about a 90% fatality rate [12].

Programs like *Counseling on Access to Lethal Means* (“CALM”) help health professionals discuss restriction confidently; one study found that 74% of providers offered lethal means counseling after completing the training [19]. Despite these benefits, providers also report feeling uncomfortable discussing suicide, lethal means, and safety planning due to lack of training, perceptions of ineffectiveness, cultural sensitivity around firearm ownership in rural areas, and fears of errors [20–24]. Overcoming provider reluctance is crucial, as research shows veterans are generally willing to discuss firearm access in trusting relationships [25], such as with their health care professionals. However, existing trainings in online/classroom formats may not provide opportunities for immersive, repeatable practice of high-stakes skills in low-risk environments. Virtual reality (VR) demonstrates feasibility in addressing these limitations by offering on-demand access to realistic skill-building opportunities without exposing patients to risk [26,27]. The VET-SAVR Home Visit module represents an initial feasibility test of this VR approach specifically for suicide risk identification and lethal means counseling with rural veterans, while future modules are planned to expand coverage of key provider training needs.

### VR training

VR uses fully immersive head-mounted displays to view a three-dimensional, computer-generated environment that simulates real-world interactions for educational and health applications ([28], p. 7). Systematic reviews and meta-analyses consistently demonstrate VR’s advantages over traditional lectures, online training, and simulations, including resource-intensive actor-based methods, for training nursing [29] and other health and mental health professionals [26,30]. By enabling repeatable practice and error correction in a low-risk environment [27,31], VR supports “learn-by-doing” skill development that would be costly or infeasible with live patients or standardized actors. While researchers have primarily focused on VR as an intervention tool for veterans, its potential as a training tool for veteran-serving healthcare professionals remains underexplored [32]. An exploratory survey of VA providers’ perceptions of VR revealed strong interest in the technology, yet its use for provider training in VA settings remains limited [33]. Rural community members (e.g., family, clergy, neighbors) also serve as informal gatekeepers who influence veterans’ help-seeking and can support safety planning, making their understanding of suicide prevention equally important.

Another line of research supports the use of VR for suicide prevention training [34–38]. Current lethal means safety training, such as Counseling on Access to Lethal Means (CALM), is delivered only in 2D online or classroom formats [39], limiting opportunities for immersive, repeatable practice in culturally sensitive conversations. No published studies have evaluated VR-based suicide prevention training specifically for professionals and community members serving rural veterans. When taken together, current research indicates an ongoing need for training interventions to strengthen the rural healthcare workforce’s ability to identify and respond to suicide risk factors in veterans. This study begins to address this gap by developing and testing a rural home-visit module within the “VET-SAVR” (Veteran Suicide Assessment in Virtual Reality) simulation focused on suicide risk identification and lethal means safety training skills.

### Research objectives

**Fig 1:**
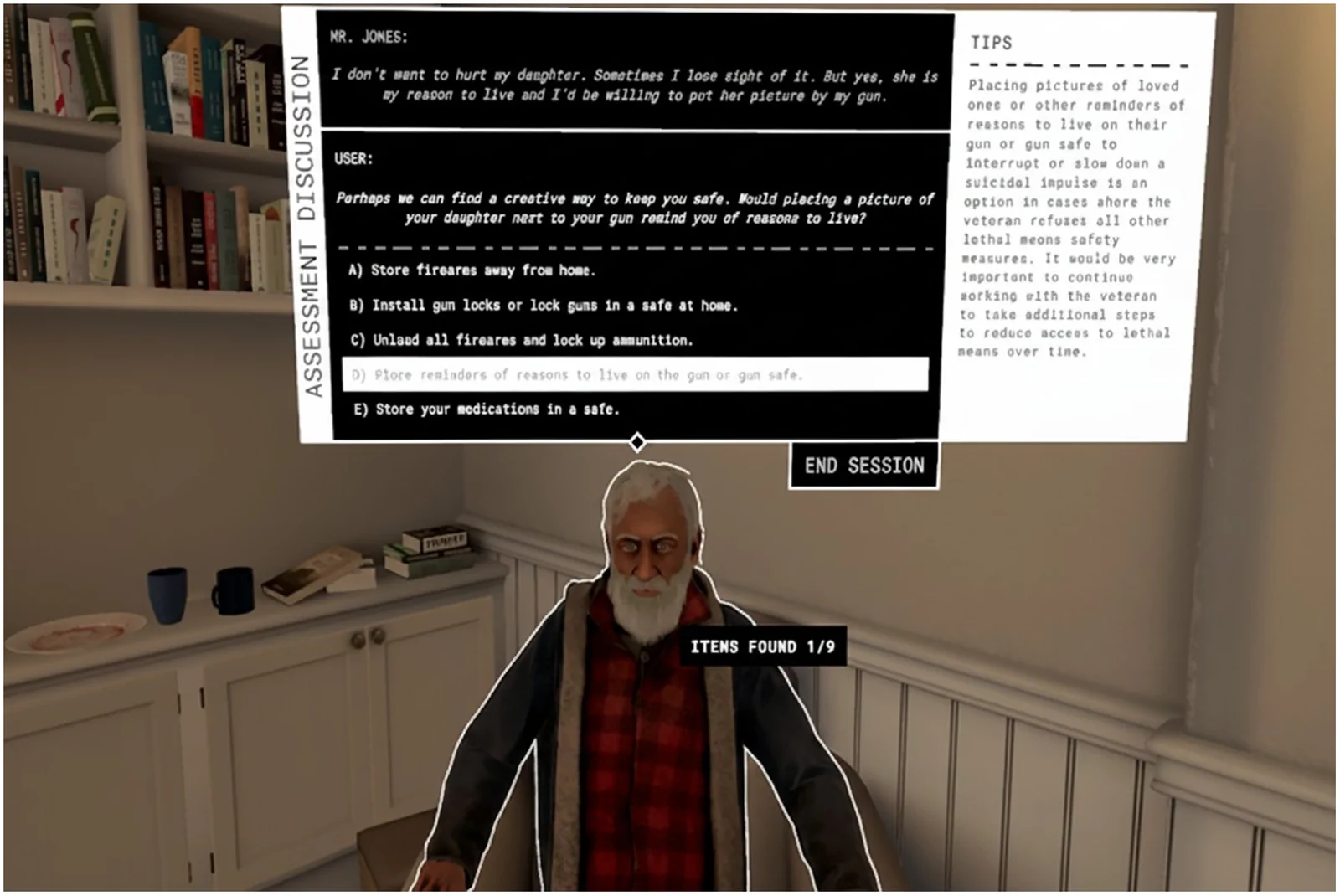
Home visit scene in VET-SAVR VR simulation. *Note.* Screenshot from the Veteran Suicide Assessment in Virtual Reality (VET-SAVR) simulation depicting a home visit with Mr. Samuel Jones, a rural veteran at risk for suicide.

In response to the need for novel, scalable suicide prevention training for professionals serving rural veterans, this mixed-methods feasibility study applied the Analysis, Design, Development, Implementation, and Evaluation (ADDIE) framework to develop and evaluate VET-SAVR, a VR-based simulation of a home visit with a rural veteran that incorporates suicide risk identification and lethal means safety counseling (see Fig 1). Primary objectives were to assess the prototype’s preliminary feasibility and acceptability by evaluating: (1) participants’ sense of presence and engagement in the VR environment, (2) perceived representativeness and realism of the simulated rural home visit, (3) usability and acceptability across diverse user groups (nursing/social work students, health professionals, and rural community members), and (4) whether participants could recall and recognize suicide risk indicators embedded in the simulation. Secondary objectives: to identify areas for refinement in prototype design.

## Materials and methods

Guided by the 5-phase ADDIE framework, a widely used model for instructional design in educational and training contexts [40], this mixed-methods feasibility study systematically developed a VR simulation prototype and evaluated its feasibility, acceptability, and user experience. Recruitment for the analysis phase (expert interviews) occurred from 10/12/2022–31/01/2023. Recruitment for the implementation and evaluation phases (VR feasibility testing with 30 participants) took place from 10/07/2023–31/10/2023. This feasibility study is reported in accordance with the Strengthening the Reporting of Observational Studies in Epidemiology (STROBE) extension for health care simulation research [41]. This study was approved by The University of Texas at Arlington Institutional Review Board (Protocol # 2022-0571). All procedures were conducted in accordance with the ethical standards outlined in the Declaration of Helsinki and applicable institutional guidelines. Prior to participation, all participants provided written informed consent after receiving information on the study’s purpose, procedures, risks, benefits, and voluntary nature, with opportunities to ask questions.

During manuscript preparation, the authors used Grammarly for language editing and Scite for literature review and citation-context checking. The authors reviewed all outputs, verified cited sources, and take full responsibility for the accuracy, originality, and integrity of the manuscript. These tools were not used to generate data, conduct analyses, or interpret findings.

### Analysis phase

In the Analysis phase, the study team recruited participants who had previously conducted home visits to rural veterans with depression and suicide risk via flyers posted online and on campus, as well as snowball sampling. All surveys were administered via QuestionPro, a secured online data collection platform. Potential participants could scan a QR code on the flyer or email the study team for a link to the online screening. After eligibility was confirmed, participants were emailed a link to the online consent and survey measures. A study team member reviewed the consent form and answered participants’ questions before the online interviews. Inclusion criteria were (a) completion of a bachelor’s or master’s degree in a healthcare or mental health field (e.g., social work, nursing, counseling) and (b) prior professional experience conducting home visits and assessing or treating veterans at risk of death by suicide.

Exclusion criteria included any medical or mental health conditions that would affect participation (e.g., active psychosis, cognitive impairment, or current suicidal ideation). To inform the development of key risk factors associated with rural veteran suicide, the research team conducted in-depth interviews with six rural healthcare providers from North Texas who are knowledgeable about providing clinical services to rural veterans with mental health concerns and have an average of 10+ years working with veterans. Participants (*M* = 54.83 years, *SD* = 11.38) were interviewed via Microsoft Teams between July 2023 and August 2023 and audio-recorded. Interviews lasted approximately 45 minutes each. Participants received a $25 Amazon e-gift card as an incentive. Table 1 presents the demographic and professional characteristics of the rural veteran health experts.

**Table 1:** Expert Panel Demographics.

Characteristic	Summary
Role	VA social work/nursing (*n* = 4); Suicide prevention/coordination (*n* = 1); Rural clinic nursing (*n* = 1)
Veteran Status	Veteran (*n* = 5); Civilian (*n* = 1)
Race/Ethnicity	White (*n* = 4); Hispanic (*n* = 2)
Gender	Male (*n* = 4); Female (*n* = 2)
Age (years)	Mean = 54.83 (*SD* = 11.38; range = 35–69)
Professional Experience (years)	Mean = 10.67 (*SD* ≈ 3.9; range = 3–15)

*Note.* Aggregated characteristics to protect participant anonymity.

No new major design-relevant themes emerged in the final interviews; however, this formative phase was not intended to provide comprehensive thematic saturation across all rural veteran care contexts. Qualitative interviews comprised 18 questions assessing rural veteran-serving professionals’ real-world practices, knowledge/training gaps, environmental contexts, suicide risk-identification cues, counseling approaches, and safety concerns. Transcripts were uploaded to QDA Miner (Version 2024) for coding. Thematic analysis was used to identify key themes, using a deductive approach triangulated with VA suicide data [10] and prior studies [9]. Through axial coding, themes were collapsed into five interconnected categories that directly informed simulation design: (1) Preparation and Team-Based Decision-Making (e.g., record review, safety planning); (2) First Impressions and Environmental Context (e.g., external home appearance, initial interactions); (3) Navigating the Home and Assessing Daily Living (e.g., signs of personal neglect, medication/food checks); (4) Lethal Means and Risk Assessment (e.g., visible firearms, direct storage discussions); and (5) Social and Emotional Health (e.g., isolation indicators, grief cues). These categories would later be used to identify environmental risk factors and interpersonal interactions commonly observed during rural home visits and to inform the VET-SAVR prototype. These are shown in Table 2.

**Table 2:** Thematic Categories Informing VET-SAVR Design.

Category	Key examples informing simulation elements
*Preparation and Team-Based Decision-Making*	Record review, assessing known risk factors (e.g., prior attempts, firearm ownership), team safety planning
*First Impressions and Environmental Context*	External home appearance (e.g., overgrown yard, disrepair), initial access/interactions
*Navigating the Home and Assessing Daily Living*	Interior neglect (e.g., overflowing trash, dirty dishes), medication/food checks
*Lethal Means and Risk Assessment*	Visible/accessible firearms (e.g., handgun on table, rifle behind door), safe storage discussions
*Social and Emotional Health*	Isolation/grief indicators (e.g., memorial items, distant family contact, loss reminders)

*Note.* Categories derived through axial coding; used to select and embed realistic cues, interactions, and decision points in the simulation.

### Design phase

In the Design phase, the research team used data collected during the Analysis phase to develop and structure the VR training simulation. A research and design team consisting of social work PhDs, nursing PhDs, video game designers, animators, social work doctoral students, and social work master’s students developed a storyboard that outlined the scenarios, environments, features, and interactions needed to reflect a home visit to a rural veteran at risk for suicide. We selected the Unity game engine for its affordability, ease of use, robust VR development capabilities, and compatibility with Meta Quest 2 [42]. The design process incorporated the thematic categories identified in the Analysis phase to ensure realism and relevance. The subject of the simulated home visit, Mr. Samuel Jones, a 75-year-old White male Vietnam-era veteran, was designed as a composite reflecting demographic profiles associated with elevated rural suicide risk (e.g., older age, White race/ethnicity, Vietnam service era) [1,2,9,43].

### Development phase

The simulation was built using *Unity* (version 2021.3), a real-time 3D development platform used to design and deploy virtual reality (VR) applications, games, and interactive simulations, serving as the underlying framework for many immersive experiences across major hardware systems, including Meta Platforms Quest devices, HTC Vive, and Sony Interactive Entertainment PlayStation VR. Simulation development was completed by a multidisciplinary research team including VR designers, animators, and a computer scientist. Content experts in veteran suicide prevention developed the script and other educational content, which the VR team incorporated into the training simulation through professional voiceovers, interactive quizzes, and educational prompts. To ensure accessibility, the VR design team created the simulation for cross-platform use, with deployment on the *Meta Quest 2* headset selected for affordability and portability. See Fig 1 for an example simulation screenshot.

Environmental cues were embedded to reflect real-world risk indicators (e.g., firearms, alcohol, financial distress, and recent loss). Interactive features enabled participants to engage with highlighted objects and review suicide-prevention tips. The development team conducted iterative rounds of playtesting to ensure platform stability and usability.

### Implementation and evaluation phases

We recruited a diverse sample of individuals serving rural communities. Potential participants included senior-level undergraduate and graduate nursing and social work students, health professionals (e.g., nurses, other medical providers, social workers, counselors), and community members. Recruitment was conducted via social media, campus flyers, and snowball sampling. We excluded individuals with vertigo, seizure disorders, pregnancy, or previous adverse VR experiences. All online screening and measures were administered using QuestionPro online survey software. Content warnings were included, and participants could withdraw at any time without penalty. Data were stored on secure, password-protected UTA servers, with identifiers anonymized through coding.

Thirty participants completed the study (10 each from the student, professional, and rural community member groups). Due to missing outcomes from one participant, some analyses include 29 participants. Participants received a 5- to 7-minute orientation to the Meta Quest 2 headset, including instructions on navigating the virtual environment and interacting with the content. Two trained graduate-level research assistants (RAs) provided technical support, guiding participants through the process. Participants practiced navigating in the simulation before initiating the virtual home visit. The RA reminded participants to observe suicide risk cues while in the virtual space. After engaging with the simulation, participants completed post-simulation standardized measures.

### Measures

Measures focused on feasibility, acceptability, and user experience, including sense of presence, perceived representativeness, and acceptability of the simulation, adverse effects, and exploratory recall of suicide risk cues.

We measured participants’ **sense of presence** using the ITC-Sense of Presence Inventory [44], which assesses the psychological experience of physical presence in a virtual environment. This 44-item self-report evaluates four domains: spatial presence (19 items), engagement (13 items), ecological validity (5 items), and negative effects (6 items). The ITC-SOPI has demonstrated strong psychometric properties [45,46]. Participants rated each item on a 5-point Likert scale, ranging from 1 (Strongly Disagree) to 5 (Strongly Agree). We computed domain scores by averaging item responses, with higher scores indicating greater presence in that dimension. Internal consistency reliability of the ITC-SOPI was evaluated separately for each domain. Cronbach’s alpha coefficients indicated excellent reliability for spatial presence (19 items; α = .90), good reliability for engagement (13 items; α = .85) and negative effects (6 items; α = .86), and acceptable reliability for ecological validity (5 items; α = .72). Our measurement approach reflected the feasibility stage of the work. Sense of presence and ecological validity are established prerequisites for effective training, while cue recall provides preliminary evidence of attention to clinically relevant information.

**Simulation acceptability and representativeness** were assessed with a single Likert-style item that asked participants to rate how well the VR scenario reflected a home visit involving a rural veteran with depression and suicidality (1 = Strongly Disagree to 5 = Strongly Agree). Two open-ended prompts followed: “What scene elements do you feel were missing that, if included, would make the VR simulation more realistic?” and “Please add any comments you’d like to make about your experience with the VR case simulation.”

To exploratively assess **recall of suicide risk cues**, participants were asked to list all risk cues they remembered from the simulation. Responses were entered in an open-ended text field and later coded during qualitative analysis.

### Procedures

All eligible participants completed an electronic consent form, along with the standard study measures and demographic information. User testing was conducted in person in a private room or office, or on the university campus. Participants put on the Meta Quest 2 headset and were given the handheld controllers. Before entering the simulation, participants received instructions from the project RAs on how to navigate and interact with objects in the virtual space. During the simulation, participants engaged with highlighted cues (such as a pill bottle on a bedroom nightstand and firearms located on a side table, or behind the front door) and answered embedded quiz questions throughout the simulation that highlighted suicide prevention considerations. After the session, RAs recorded session duration, performance on embedded quizzes, and wrote observational notes. Embedded quiz responses were logged to confirm task engagement and simulation functionality but were not analyzed as learning outcomes in the feasibility phase. Participants then completed the ITC-SOPI scale, answered questions about the simulation’s representativeness, and submitted open-ended feedback.

### Data analysis

Both quantitative and qualitative analyses focused on indicators of feasibility, acceptability, and user experience. The research team conducted quantitative analyses in IBM SPSS Statistics version 29, employed qualitative content analysis for open-ended responses, and completed coding in Microsoft *Excel*.

For the quantitative analysis, Shapiro–Wilk tests indicated approximate normality for most ITC-SOPI subscales; however, ecological validity scores deviated significantly from normality. Given the small sample size and ordinal response format, nonparametric tests were used for group comparisons. Data were determined as missing completely at random and handled using listwise deletion for variables with incomplete data. Pearson or Spearman rank-order correlations were used, as appropriate, to examine relationships among ITC-SOPI domains, prior VR experience, and time spent in the VR environment. Alpha levels were set at .05 for all significance testing.

Group differences in ITC-SOPI subscales (spatial presence, engagement, ecological validity, and negative effects) were examined using Kruskal–Wallis tests. Effect sizes were calculated using epsilon-squared (ε²) and interpreted according to Tomczak and Tomczak’s [47] criteria for small (ε² ≈ .01), medium (ε² ≈ .06), and large (ε² ≈ .14) effects.

For the qualitative analysis, the research team analyzed open-ended participant feedback using thematic analysis. To ensure rigor, the team developed a coding framework, used multiple coders, conducted peer debriefings, and maintained an audit trail. The lead author and a graduate RA independently coded participants’ narrative responses using an inductive approach [48], met to compare codes, and iteratively refined a shared codebook. A third coder (a doctoral student) resolved discrepancies and verified subthemes, which were then collapsed into broader thematic categories.

For an exploratory examination of recalled suicide risk indicators, we employed a summative content analysis approach [49], in which specific environmental and behavioral cues mentioned by participants were tallied and reported as frequencies and percentages to characterize cue salience. Rigor was supported through multiple coders, peer debriefing, and maintenance of an audit trail.

## Results

Results are organized by feasibility domains: participant characteristics; implementation feasibility; user experience (presence and adverse effects); exploratory recall of suicide risk indicators; and acceptability.

### Participant characteristics

Participants had a mean age of 41.27 years (*SD* = 16.00, range = 21–82); 60.0% were female (*n* = 18) and 40.0% male (*n* = 12). Participants’ racial/ethnic composition was as follows: White (53.3%, *n* = 16), Hispanic (33.3%, *n* = 10), Black/African American (10.0%, *n* = 3), and Asian (3.3%, *n* = 1). Nearly 27% of participants were veterans (*n* = 8) (see Table 3).

**Table 3:** Participant Demographics.

Variable	*n*	%	Mean (SD)	Range
Age			41.27 (16.00)	21–82
**Age Group**				
18–30 years	9	30.0		
31–50 years	12	40.0		
51+ years	9	30.0		
**Gender**				
Male	12	40.0		
Female	18	60.0		
**Race/Ethnicity**				
Asian	1	3.3		
Black/African American	3	10.0		
Hispanic	10	33.3		
White	16	53.3		
**Veteran Status**				
Yes	8	26.7		
No	22	73.3		
**Military Family Member (Parent, Spouse, Child, Other)**				
Yes	21	70.0		
No	9	30.0		
**Participant Group**				
Students	10	33.3		
Professionals	10	33.3		
Rural Community Members	10	33.3		

### Implementation feasibility

All 30 participants completed the simulation. Time spent in the simulation varied by group: students (*n* = 10) averaged 13.03 minutes (*SD* = 3.69), professionals (*n* = 10) averaged 16.02 (*SD* = 2.34), and community members (*n* = 10; one missing) averaged 14.60 (*SD* = 3.33) (see S1 Table). Despite overall low levels of adverse effects, some participants reported mild adverse effects (e.g., nausea).

### User experience: Sense of presence

Participants’ sense of presence was assessed using the ITC-SOPI. Across participant groups, mean engagement scores ranged from 3.64 to 4.02, spatial presence from 3.40 to 3.70, and ecological validity from 3.56 to 3.92, while negative effects remained relatively low (2.35–2.63) (see S1 Table).

Spearman correlations revealed a significant negative association between prior VR experience and negative effects (ρ = −.370, p = .044). Time spent in the simulation was positively correlated with negative effects (r = .397, p = .033) (see S2 Table). Kruskal–Wallis tests indicated no significant group differences for engagement, χ²(2) = 2.55, *p* = .280, ε² = .088; spatial presence, χ²(2) = 1.80, *p* = .406, ε² = .062; ecological validity, χ²(2) = 0.84, *p* = .657, ε² = .029; or negative effects, χ²(2) = 0.46, *p* = .794, ε² = .016 (see Table 4).

**Table 4:** Group Differences (Kruskal-Wallis).

Variable/Subscale	χ² (df = 2)	*p*-value	*ε*²
ITC-SOPI Engagement	2.55	.280	.088
ITC-SOPI Spatial Presence	1.80	.406	.062
ITC-SOPI Ecological Validity	0.84	.657	.029
ITC-SOPI Negative Effects	0.46	.794	.016
Number of Objects Recalled	3.09	.214	.110

*Note.* ITC-SOPI = ITC-Sense of Presence Inventory (adapted from Lessiter, Freeman, Keogh, and Davidoff [44]). Tests compared Students (*n* = 10), Professionals (*n* = 10), and Rural Community Members (*n* = 10 for ITC-SOPI subscales; *n* = 9 for Number of Objects Recalled due to one missing case). ε² effect sizes follow Tomczak and Tomczak [47]: small ≈ .01, medium ≈ .06, large ≈ .14. No significant group differences were found (*p* > .05).

### Exploratory recall of suicide risk indicators

Participants recalled an average of 8.55 objects (*SD* = 0.74, range = 7–9) out of 9, suggesting strong attention to embedded cues. Kruskal–Wallis tests showed no significant group differences in the number of objects recalled, χ²(2) = 3.09, *p* = .214, ε² = .110 (see Table 4). Detailed statistics and correlations are provided in Table 4 and S2 Table.

**Fig 2:**
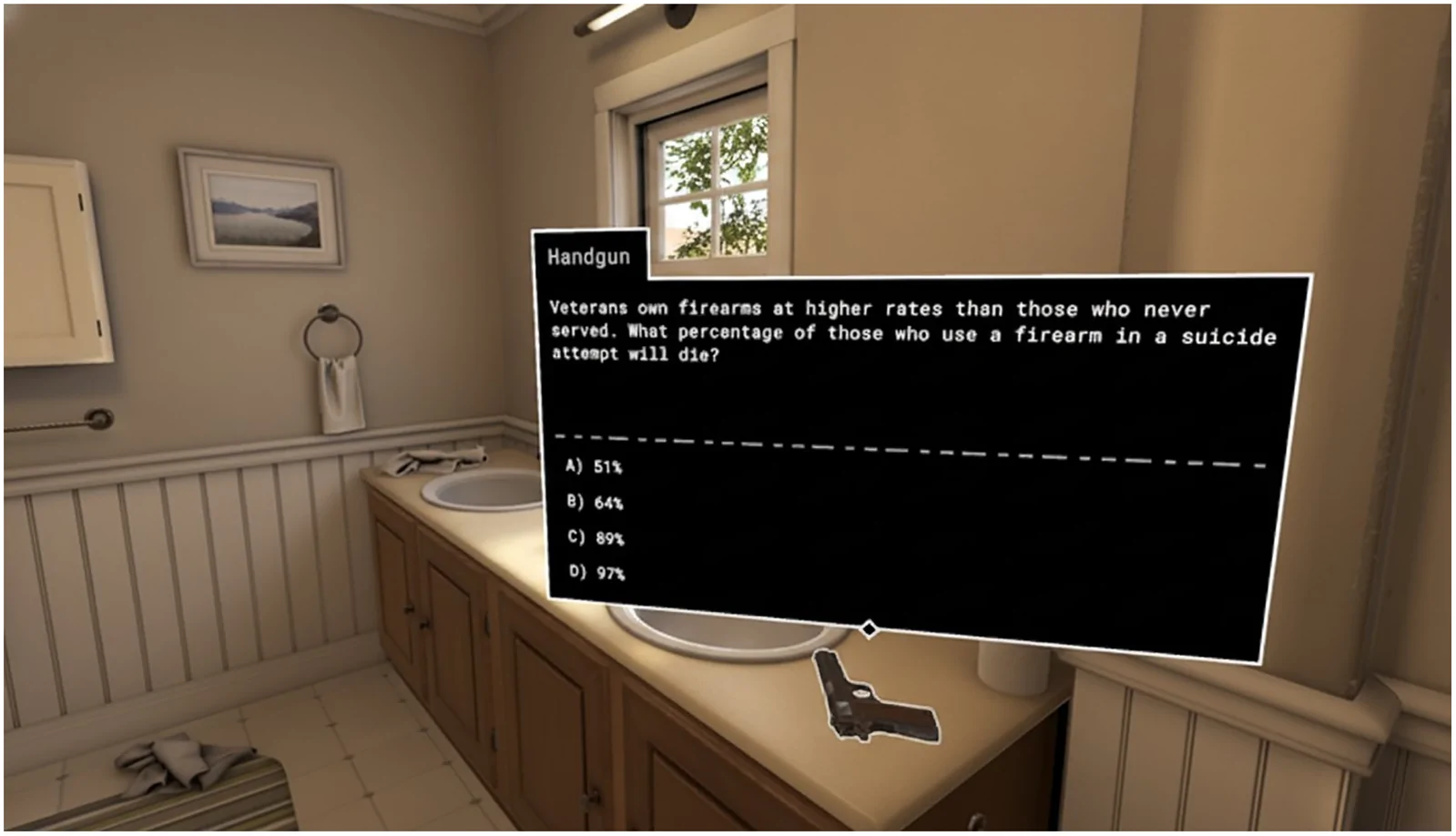
Participant recall of suicide risk elements in the VET-SAVR simulation. *Note.* Screenshot from the VET-SAVR simulation depicting an unsecured firearm, a frequently recalled element indicative of suicide risk (93.3%, *n* = 28).

Examination of recalled scene elements (e.g., unsecured firearm; see Fig 2) indicated differential cue salience. Firearms (93.3%; *n* = 28) and alcohol (83.3%; *n* = 25) were cited most frequently, reflecting high visibility of prominent lethal means cues. Many participants also recalled medication or drugs (76.7%; *n* = 23) and final arrangements (e.g., will; 63.3%; *n* = 19), suggesting visibility of contextual risk indicators. Participants recalled behavioral signs moderately often, including giving away belongings (56.7%; *n* = 17) and poor appetite (53.3%; *n* = 16). In contrast, subtler cues, like books on death (6.7%; *n* = 2) and the veteran’s appearance or age (3.3%; *n* = 1), were rarely mentioned. (See Table 5).

**Table 5:** Frequency of Recalled Suicide Risk Indicators.

Category	Count	%
**Lethal Means**		
Firearms	28	93.3
Alcohol (Substance misuse)	25	83.3
Medication/Drugs/Opioids	23	76.7
**Behavioral/Risk Indicators**		
Will/Financial Arrangements	19	63.3
Giving Away Belongings/Pet	17	56.7
Poor Appetite/Expired Food	16	53.3
Suicide Note/Goodbye Letters	14	46.7
Death of Spouse/Anniversary	13	43.3
Isolation	6	20.0
Books on Death or Dying	2	6.7
Appearance or Age	1	3.3
**Other**		
Picture of Loved Ones/Dog	7	23.3
PTSD/Depression	2	6.7

*Notes.* Values reflect the open-ended recall of environmental cues by 30 participants, who could recall multiple elements. Categories are grouped into Lethal Means, Behavioral/Risk Indicators, and Other to emphasize patterns in interpretation. Percentages indicate the proportion of participants who mentioned each element.

### Acceptability

Most VR participants agreed that the simulation accurately represented a rural home visit involving suicidality and depressive symptoms. Qualitative feedback (*n* = 25) indicated that most frequently reported theme was a positive VR experience (*n* = 9; 36%), whereas physical discomfort was least frequently reported (*n* = 2; 8%). See S3 Table for themes from content analysis.

## Discussion

This ADDIE-guided mixed methods pilot study systematically developed and conducted a feasibility evaluation of a VR simulation designed to replicate a home visit with a rural veteran at risk for suicide, embedding suicide risk identification and exposure to lethal means safety considerations. Regarding users’ sense of presence, participants reported high engagement and a moderate sense of spatial presence within the VR environment. Recalled scene elements provided exploratory insight into cue salience within the simulation. Participants most frequently recalled firearms, alcohol, medication or drugs, and final arrangements (e.g., will), suggesting high perceptual salience of these prominent cues in portraying suicidality [50]. Less frequently recalled elements, such as isolation cues and books about death, suggest the need to strengthen visual and educational emphasis in future iterations. Planned refinements to improve detection of subtler cues (e.g., isolation, books about death) include guided reflection prompts, staged cue exposure, structured debriefing, and interactive dialogue with the veteran character. Participants’ weak recall of social isolation as a cue is particularly notable, given its well-established role as a significant contributor to suicide risk [50]. The pattern of strong recall of visually salient cues but weaker recognition of behavioral/contextual indicators suggests VR-based training, through immersive environmental exposure and guided practice, may support attention to both obvious and subtle risk indicators. Most of our study participants agreed that the VR simulation accurately depicted a rural home visit involving suicidality and depressive symptoms.

Participants’ experiences with the simulation provided preliminary evidence of feasibility and acceptability. Participants recalled 8.55 of 9 embedded suicide risk indicators (SD = 0.74), with strongest recall of high-salience visual cues (firearms: 93.3%; alcohol: 83.3%) and lower recall of contextual or behavioral cues (isolation: 20.0%; books about death/dying: 6.7%). This pattern suggests the prototype appears to direct attention to salient risk factors but may benefit from design refinements to enhance encoding of subtler indicators. All participants completed the simulation; however, average session times varied across groups. Time spent in the simulation was positively associated with negative effects, and prior VR experience was negatively associated with them, suggesting that prolonged exposure and VR-naïveté may increase discomfort, a pattern that warrants further examination among VR-naïve users (i.e., those with very little or no prior use). These observations suggest that user characteristics and VR system configuration, including head-mounted display specifications (e.g., Meta Quest 2), may influence the likelihood and severity of adverse effects [51]. While participants deemed the simulation feasible and acceptable, suggested improvements included clearer navigation cues and more responsive interactions with the veteran avatar. Future studies could examine strategies to improve VR safety and comfort, such as shorter initial exposure periods, scheduled breaks, and adjustable visual or motion settings, particularly for VR-naïve users.

### Limitations

This feasibility study has several important limitations. The three-user-group sample was recruited through convenience and snowball sampling, limiting generalizability; students and community members may not be intended end-users of a professional training tool. Additionally, the cue recall task was prompted (participants were instructed to attend to suicide risk indicators), so recall rates may not reflect naturalistic detection of these cues in clinical practice. Subgroup comparisons (9–10 per group) are underpowered and should be considered preliminary and exploratory. The veteran composite reflected demographic profiles associated with elevated rural suicide risk (e.g., older age, White race/ethnicity, Vietnam era; [1]); future iterations of the simulation development plan will expand the diversity of rural veteran patients (e.g., younger veterans, women, racial/ethnic minorities) to enhance inclusivity and representativeness. Participants’ prior suicide prevention training and clinical home visit experience were not collected and therefore could not be examined as moderators of cue recall, perceived realism, or acceptability. Future studies should capture these characteristics, particularly for student and professional trainees. The small sample size also precluded statistical adjustment for prior VR experience and necessitated the use of nonparametric statistics. Overall, adverse effect levels were low and did not differ significantly across groups, although they were associated with longer exposure and less prior VR experience. Some participants reported mild discomfort during longer sessions, suggesting potential usability limitations of the Meta Quest 2 headset. Its Fresnel lenses and lower resolution may contribute to discomfort compared with the upgraded Meta Quest 3 [51] or other head-mounted displays, such as the HTC Vive Pro or the Pico Neo 3. The qualitative expert sample used to inform the development was small (*n* = 6); however, the findings were triangulated with the existing literature.

### Implications for practice and training

Many community-based and rural health professionals lack specialized training in veteran mental health and suicide prevention [15]. Immersive VR offers flexibility for prototype development and, pending further testing, may support scalable, on-demand delivery options for training contexts [27], potentially expanding access to veteran-specific training for providers working both within and outside the Veterans Administration healthcare system. For nursing education and rural practice, VET-SAVR offers a simulation tool that can easily be integrated into curricula and continuing education, equipping nurses (who often serve as frontline gatekeepers for rural veterans) with immersive exposure to suicide risk cues and lethal means counseling, all at no risk to an actual veteran who may be contemplating suicide. As such, VR simulations have the potential to be used by a broader range of healthcare professionals, students, and community gatekeepers supporting rural veterans. By replicating high-stakes, emotionally charged scenarios, VR-based simulations may help providers prepare to recognize acute crises and to explore safety plans in real time. Moreover, tailored scenarios that reflect veterans’ cultural and psychosocial experiences may foster empathy and sensitivity to their unique risk factors and barriers to help-seeking.

### Implications for future research

Future research should evaluate the long-term effects of VR-based suicide-prevention exposure (compared with training methods such as workshops and/or role-plays) and whether VR-based training can sustain gains in knowledge, self-efficacy, decision quality, counseling skill and transfer outcomes across a broad range of provider types and community members who provide care for rural veterans. In addition, research should investigate ways to enhance the “hands-on” experience of VR-based training. Evaluating different head-mounted display (HMD) options and ergonomic modifications to reduce fatigue and discomfort during extended training sessions may help make VR more acceptable to users. More importantly, future research should go beyond establishing the feasibility of VR-based training to examine whether VR-exposed providers demonstrate improvements in clinical outcomes, such as consistently identifying suicidal risk, developing effective safety plans, and linking participants to subsequent care. In addition, economic evaluations will be needed to assess whether VR-based training is a cost-effective and scalable option relative to other training models, particularly in rural and limited-resource settings. Future iterations should evaluate VET-SAVR specifically within nursing and social work programs to measure impacts on clinical confidence and competency in veteran-specific suicide prevention. Overall, the simulation demonstrates preliminary feasibility and acceptability, warranting ongoing refinement of both content and hardware design. A subsequent efficacy trial is needed to evaluate whether this VR approach improves participant knowledge, confidence, or counseling behavior in suicide prevention.

## Conclusion

This mixed-methods feasibility study provides preliminary evidence that a VR-based home visit simulation is feasible and acceptable for training rural health professionals in suicide risk identification and lethal means safety counseling. Participants reported high engagement, moderate spatial presence, and accurate recall of salient risk cues, suggesting the simulation effectively replicates the key elements of a high-stakes encounter with a rural veteran at risk for suicide. Some aspects require further refinement and testing, notably mild adverse effects and navigational challenges in the simulation. Findings preliminarily support further development of VET-SAVR as a prototype for further development to address critical training gaps in veteran suicide prevention, particularly for frontline health professionals and community workers in rural settings. Continued iterative development and rigorous evaluation of training outcomes and clinical impact are warranted for the simulation to reach its potential in reducing rural veteran suicide risk.

## Supporting information

S1 Table Feasibility and user experience outcomes by participant group. (DOCX)

S2 Table Correlations among VR exposure variables and ITC-SOPI presence domains. (DOCX)

S3 Table Themes from open-ended participant feedback on the VR simulation experience. (DOCX)
