# Supplementary material for: ADDIE-guided development and pilot feasibility evaluation of a prototype VR training simulation for suicide prevention among rural U.S. veterans
Source: PLoS One. 2026 Aug 24;21(8):e0345819. doi: 10.1371/journal.pone.0345819 (PMC13502552; doi:10.1371/journal.pone.0345819)
Supplement: Supplementary file 1 [file pone.0345819.s001.docx]

**S1 Table. Feasibility and user experience outcomes by participant group.**

| **Variable** | **Students**  **(*n* = 10)** | | **Professionals (*n =* 10)** | | **Rural community members (*n* = 9)** | |
| --- | --- | --- | --- | --- | --- | --- |
|  | *M (SD)* | IQR | *M (SD)* | IQR | *M (SD)* | IQR |
| VR experience | 2.20 (1.14) | 1.50 | 1.90 (1.20) | 2.00 | 2.20 (1.40) | 3.00 |
| SOPI negative effects | 2.55 (0.78) | 1.29 | 2.63 (0.97) | 1.71 | 2.35 (0.76) | 0.62 |
| SOPI spatial presence | 3.70 (0.36) | 0.28 | 3.40 (0.73) | 0.91 | 3.59 (0.47) | 0.72 |
| SOPI ecological validity | 3.92 (0.63) | 0.65 | 3.56 (0.84) | .90 | 3.88 (0.34) | 0.30 |
| SOPI engagement | 4.02 (0.49) | 0.79 | 3.63 (0.51 | 0.52 | 3.82 (0.38) | 0.50 |
| Number of objects found | 8.30 (0.82) | 1.00 | 8.80 (0.63) | 0.00 | 8.56 (0.73) | 1.00 |
| Time in VR (minutes) | 13.03 (3.69) | 3.98 | 16.02 (2.34) | 3.91 | 14.60 (3.33) | 5.65 |

*Notes. M* = Mean, *SD* = Standard Deviation, IQR = interquartile range. For Time in VR, means are reported in minutes (with SD and IQR converted from seconds). VR experience level was measured on a 4-point scale (1 = none, 2 = low, 3 = moderate, 4 = high). SOPI variables (spatial presence, engagement, ecological validity, negative effects) were assessed using the ITC-Sense of Presence Inventory (ITC-SOPI; adapted with permission from Lessiter, Freeman, Keogh, and Davidoff [44]) and measured on a 5-point Likert scale (1 = strongly disagree, 5 = strongly agree). One rural community participant was excluded from analyses of Time in VR and Number of Objects Found due to missing data (*n* = 9).
