# Supplementary material for: ADDIE-guided development and pilot feasibility evaluation of a prototype VR training simulation for suicide prevention among rural U.S. veterans
Source: PLoS One. 2026 Aug 24;21(8):e0345819. doi: 10.1371/journal.pone.0345819 (PMC13502552; doi:10.1371/journal.pone.0345819)
Supplement: Supplementary file 2 [file pone.0345819.s002.docx]

**S2 Table. Correlations among VR exposure variables and ITC-SOPI presence domains.**

| **Variables** | **Pearson’s *r*** | **Spearman’s *ρ*** | ***p*-value** | **95% CI** |
| --- | --- | --- | --- | --- |
| **VR Experience Level and Presence (N = 29)** |  |  |  |  |
| Spatial Presence | N/A | .191 | .313 | [−.199, .545] |
| Engagement | N/A | .148 | .436 | [−.234, .487] |
| Ecological Validity | N/A | .353 | .056 | [.004, .659] |
| Negative Effects | N/A | −.370 | .044 | [−.708, .014] |
| **VR Time Taken and Presence (N = 29)** |  |  |  |  |
| Spatial Presence | .203 | N/A | .424 | [−.176, .422] |
| Engagement | .313 | N/A | .401 | [−.123, .468] |
| Ecological Validity | .294 | N/A | .584 | [−.139, .333] |
| Negative Effects | .220 | N/A | .033 | [−.034, .723] |
| **VR Objects Found and Presence (N = 29)** |  |  |  |  |
| Spatial Presence | −.230 | N/A | .951 | [−.406, .416] |
| Engagement | .194 | N/A | .354 | [−.640, .390] |
| Ecological Validity | .084 | N/A | .346 | [−.575, .380] |
| Negative Effects | −.202 | N/A | .715 | [−.235, .333] |

*Notes.* **ITC-SOPI** = ITC-Sense of Presence Inventory (adapted with permission from Lessiter, Freeman, Keogh, and Davidoff [43]); **VR** = virtual reality. Spearman’s rho was used for correlations involving the ordinal variable “Prior VR Experience” and the non-normally distributed ITC-SOPI Ecological Validity subscale. Pearson’s *r* was used for all other continuous variables. One participant had missing data for simulation time and objects recalled (*N* = 29 for those correlations). All 95% confidence intervals are bias-corrected percentile intervals based on 1,000 bootstrap samples.
