# Supplementary material for: ADDIE-guided development and pilot feasibility evaluation of a prototype VR training simulation for suicide prevention among rural U.S. veterans
Source: PLoS One. 2026 Aug 24;21(8):e0345819. doi: 10.1371/journal.pone.0345819 (PMC13502552; doi:10.1371/journal.pone.0345819)
Supplement: Supplementary file 3 [file pone.0345819.s003.docx]

**S3 Table. Themes from open-ended participant feedback on the VR simulation experience.**

| **Theme** | **Percentage (*%*)** | **Observations (n)** |
| --- | --- | --- |
| Positive VR experience | 36 | 9 |
| Need for more interaction/information | 28 | 7 |
| Technical issues with VR | 24 | 6 |
| Educational value | 20 | 5 |
| Suggestions for improvement | 20 | 5 |
| Physical discomfort | 8 | 2 |

*Note.* Themes were derived through content analysis of open-ended responses (n = 25 participants) from VR participants regarding the overall VR simulation experience, feasibility, and acceptability. Percentages indicate the proportion of participants who mentioned each theme; multiple themes may be endorsed by a single participant.
